# Translating Systems Medicine Into Clinical Practice: Examples From Pulmonary Medicine With Genetic Disorders, Infections, Inflammations, Cancer Genesis, and Treatment Implication of Molecular Alterations in Non-small-cell Lung Cancers and Personalized Medicine

**DOI:** 10.3389/fmed.2019.00233

**Published:** 2019-10-29

**Authors:** Julian Pinsolle, Anne McLeer-Florin, Matteo Giaj Levra, Florence de Fraipont, Camille Emprou, Elisa Gobbini, Anne-Claire Toffart

**Affiliations:** ^1^Department of Pneumology, CHU Grenoble Alpes, Grenoble, France; ^2^Medicine Faculty, Université Grenoble Alpes, Grenoble, France; ^3^Departement of Pathological Anatomy and Cytology, Pôle de Biologie et Pathologie, CHU Grenoble Alpes, Grenoble, France; ^4^UGA/INSERM U1209/CNRS 5309-Institute for Advanced Biosciences - Université Grenoble Alpes, Grenoble, France; ^5^Department of Biochemistry, Molecular Biology and Environmental Toxicology, CHU Grenoble Alpes, Grenoble, France; ^6^Cancer Research Center Lyon, Centre Léon Bérard, Lyon, France

**Keywords:** non-small-cell lung cancer, molecular alterations, next-generation sequencing, liquid biopsy, ALK rearrangement, EGFR mutation, tyrosine kinase inhibitors

## Abstract

Non-small-cell lung cancers (NSCLC) represent 85% of all lung cancers, with adenocarcinoma as the most common subtype. Since the 2000's, the discovery of molecular alterations including epidermal growth factor receptor (*EGFR*) mutations and anaplastic lymphoma kinase (*ALK*) rearrangements together with the development of specific tyrosine kinase inhibitors (TKIs) has facilitated the development of personalized medicine in the management of this disease. This review focuses on the biology of molecular alterations in NSCLC as well as the diagnostic tools and therapeutic alternatives available for each targetable alteration. Rapid and sensitive methods are essential to detect gene alterations, using tumor tissue biopsies or liquid biopsies. Massive parallel sequencing or Next Generation Sequencing (NGS) allows to simultaneously analyze numerous genes from relatively low amounts of DNA. The detection of oncogenic fusions can be conducted using fluorescence *in situ* hybridization, reverse-transcription polymerase chain reaction, immunohistochemistry, or NGS. *EGFR* mutations, *ALK* and *ROS1* rearrangements, *MET* (MET proto-oncogenereceptor tyrosine kinase), *BRAF* (B-Raf proto-oncogen serine/threonine kinase), *NTRK* (neurotrophic tropomyosin receptor kinase*)*, and *RET* (ret proto-oncogene) alterations are described with their respective TKIs, either already authorized or still in development. We have herein paid particular attention to the mechanisms of resistance to EGFR and ALK-TKI. As a wealth of diagnostic tools and personalized treatments are currently under development, a close collaboration between molecular biologists, pathologists, and oncologists is crucial.

## Key Concepts

- Massive parallel sequencing, known as NGS, could be particularly suited to multiplexed assessment of driving somatic alterations.
- Gene rearrangements are rare events, with no single detection technique shown to be 100% sensitive and specific. NGS platforms present several key advantages over FISH, IHC, or RT-PCR.
- Despite an initial response to EGFR inhibitors, patients develop acquired resistance and relapse. The most frequent mechanism of resistance (50–60%) is the T790M mutation in exon 20.
- The strategy of sequencing ALK inhibitors was associated with a median overall survival of 81–89.6 months.

## Introduction

Lung cancer is the leading cause of cancer mortality, accounting for 1,600,000 annual deaths worldwide and results in a 5-years survival rate of 19%. Non-small-cell lung cancers (NSCLC) represent 85% of all lung cancers, with adenocarcinoma as the most common subtype. Lung cancers are often diagnosed at an advanced stage, with worse prognosis (1).

In the 2000's, the discovery of *epidermal growth factor receptor* (*EGFR)* mutations and a specific tyrosine kinase inhibitor (TKI) gefitinib, led to the development of personalized treatments for NSCLC (2). Over the last 2 decades, other NSCLC genetic alterations have been described, such as *anaplastic lymphoma kinase (ALK), ROS proto-oncogene 1 (ROS1), rearranged during transfection proto-oncogene gene (RET), neurotrophin kinase (NTRK)* gene rearrangements, *B-Raf proto-oncogene, serine/threonine kinase (BRAF)* mutations*, MET proto-oncogene (MET)* amplification, as well as *MET* exon 14 skipping (3–9). These genes encode tyrosine kinase receptors (TKR) and their alterations have been shown to induce their constitutive activation, thereby driving carcinogenesis through intra-cellular signaling. Their inhibition by specific TKIs leads to cellular apoptosis. Several EGFR-, ALK,- or ROS1-inhibitors, currently used in clinical settings, have considerably improved NSCLC patients' overall response rates (ORR), progression-free survival (PFS), and overall survival (OS), compared to standard chemotherapy.

Consequently, molecular profiling of patients with advanced NSCLC is now systematically performed in lung adenocarcinoma patients, with a targetable molecular alteration found in 15–20% of Caucasian patients (10). In clinical practice, different molecular diagnostic tools are employed for detecting these alterations, such as immunochemistry (IHC), fluorescent *in situ* hybridization (FISH), and DNA- or RNA-based sequencing.

This review focuses on the biology of molecular alterations in NSCLC, and on diagnostic tools and therapeutic alternatives for each targetable alteration (Table 1).

**Table 1:** Known oncogenic drivers with sensibility to targeted therapies in NSCLCs (7, 10–13).

**Oncogenic drivers**	**Prevalence in NSCLCs (%)**	**EMA-approved tyrosine kinase inhibitors**
*EGFR* mutations	11	Gefitinib, erlotinib, afatinib, osimertinib
*ALK* rearrangements	5	Crizotinib, ceritinib, alectinib, brigatinib, lorlatinib
*MET* exon 14 mutations	3–4	–
*MET* amplifications	2–4	–
*BRAF* V600E mutations	1–2	Dabrafenib + trametinib
*RET* rearrangements	1–2	–
*NTRK* rearrangements	0.1–1	–

*EMA, European Medicine Agency; NSCLC, non small cell lung cancer*.

## Biology of EGFR and ALK alterations in NSCLC

### EGFR Mutations

*EGFR*, a member of the EGFR family, is a TKR that activates multiple signal transduction pathways. In NSCLC, *EGFR* mutations correspond to somatic gain-of-function mutations, occurring within the tyrosine kinase domain (14). These alterations commonly consist of in-frame deletions in exon 19 (45–50%) or the L858R substitution in exon 21 (40–45%). Uncommon alterations represent 10% of *EGFR* mutations, which induce a heterogeneous response to EGFR-TKIs, along with a poorer prognosis than that of more frequent mutations (15, 16). Exon 18 G719X are the most frequent alterations in this subgroup, accounting for 28% of all rare mutations, is followed by exon 21 L861Q (16–35% of cases) and exon 20 S768I (5% of cases) alterations (17, 18).

*EGFR* mutations are identified in 11% of NSCLCs and in 44% of non-smoker patients (10). These alterations are mainly observed in non-smoking, Asian, and female patients.

### ALK Rearrangements

The *ALK* gene is located on the short arm of chromosome 2 and encodes a TKR, member of the insulin receptor family. The ALK receptor is activated by two ligands: FAM150A and FAM150B (19, 20). The precise role of the ALK protein in humans is still unknown, whereas the *ALK* gene plays a role in mice's neuronal development and testicular function (21, 22). The ALK protein is physiologically not expressed in the lung tissue. These alterations correspond to either an inversion or translocation, leading to a fusion between the 3′ portion of *ALK* and 5′ portion of a partner gene. These fusion genes encode a fusion protein that activates signaling pathways (e.g., PI3K-AKT, JAK-STAT, MAPK pathways), promoting carcinogenesis (23).

In NSCLC, several fusion partners have been described. The most common of these is *echinoderm microtubule-associated protein like-4* (EML4), located on the short arm of chromosome 2 but separated from *ALK* by 12 Mb (24). The breakpoint site in the partner gene can occur within different exons, thereby defining the fusion variant. Consequently, different fusion variants have been identified, the most frequent being *EML4(13)-ALK(20)*, which consists of a fusion between exon 13 of *EML4* and exon 20 of *ALK*. This is also known as *EML4-ALK* variant 1. Three characteristics are shared by the reported *ALK* fusion variants. First, the entire *ALK* kinase domain is conserved. Second and third, the partner promoter and its oligomerization domain are both preserved, inducing an aberrant expression and constitutive activation of the fusion protein (25). As a result, levels of ALK fusion protein expression, along with invasion or proliferation capacities of the tumor cells, could depend on the nature of the fusion variant (26). Moreover, the breakpoint site within the gene partner could affect protein stability and, thus, treatment sensitivity (27, 28). Some clinical data showed a link between the variant nature and the TKI response (29).

*ALK* rearrangements are rare. They are identified in 2–7% of NSCLCs and 15% of non-smoker patients, mainly in young patients (3, 10, 30, 31).

## Diagnostic Tools for Detecting Molecular Alterations in a Clinical Setting

Accurate and timely detection of these oncogenic alterations, has been proven crucial, as the products of these alterations can be targeted by a growing list of inhibitors, leading to tumor growth inhibition and regression.

### Methods for Genotyping Tumor Tissues or Liquid Biopsies

Genotyping of somatic genetic alterations has become routine practice for patient management from baseline to disease's progression following targeted therapies. Lung cancers are predominantly diagnosed through biopsy, but the quantity of tumor cells in each biopsy varies, largely depending on tumor cellularity and size of the specimen acquired. Furthermore, most tumor tissues are preserved in formalin-fixed paraffin-embedded (FFPE) blocks, which crosslink the nucleic acids, thereby resulting in fragmented DNA. Finally, *EGFR* testing should be available as soon as possible to enable first-line therapy using EGFR antagonists. Therefore, genotyping of NSCLC biopsies requires rapid and sensitive technologies that only require a small amount of input DNA.

While Sanger sequencing, the gold standard technology that characterizes a mutation in hereditary disease, proves to be not sensitive enough, numerous alternative sequencing methods have been developed and validated for the clinic (Table 2). In France, a multicenter study named ERMETIC (Evaluation of EGFR Mutation Status for the administration of EGFR-TKIs in Non-Small Cell Lung Carcinoma) was conducted to validate numerous methods based on restriction enzyme analysis, allele-specific amplification, single-base extensions, fluorogenic allele-specific oligonucleotide hybridization probe, pyro-sequencing, or high-resolution melting (33).

**Table 2:** Methodologies for detecting mutations [modified according to Diaz and Bardelli (32)].

**Technique**	**Sensitivity**	**Optimal application**	**Detection of known hot spot/other mutations**
Sanger sequencing	>10%	Tumor tissue	Yes/Yes
Pyrosequencing	5–10%	Tumor tissue	Yes/No
Next-generation sequencing	2%	Tumor tissue	Yes/Yes
Quantitative PCR	1%	Tumor tissue	Yes/No
ARMS	0.1%	Tumor tissue, ctDNA	Yes/No
BEAMing, Digital PCR	0.01%	ctDNA, rare variants in tumor tissue	Yes/No
TAM-Seq	0.01%	ctDNA, rare variants in tumor tissue	Yes/Yes

*ARMS, amplification refractory mutation system; ctDNA, circulating tumor DNA; BEAMing, beads, emulsification, amplification and magnetics binding; TAM-seq, tagged-amplicon deed sequencing; PCR, polymerase chain reaction*.

All these methods are single-gene approaches and must be multiplied by a rapidly growing number of predictive markers. Massive parallel sequencing, also known as next-generation sequencing (NGS), is particularly well-suited to the multiplexed assessment of somatic alterations (34). Associated with panel development, bioinformatic supports permit to evaluate the sequencing coverage and to characterize and annotate not only known hot spot mutation but also other molecular anomalies [non-hot-spot mutations, gene copy number (CNV)]. For clinical applications, targeted sequencing of a limited set of essential genes has so far proven to be the most practical approach. Indeed, targeted panels offer the advantage of high depth (>300X), as well as high overall exon coverage (>99%). These two quality markers secure the sensitivity of NGS at around 2%. Thus, the choice of a specific clinical NGS assay requires careful consideration of panel size, inclusion of appropriate markers, the ability to detect multiple genomic aberration types, and compatibility with a low quality and quantity of nucleic acids (35). This choice is also dependent of the analysis's cost. Sabatine et al. (36) have demonstrated that NGS price varies from about 600–3,400$ depending on the number of genes including in the panel. Recently, Simarro et al. (37) have compared cost of conventional molecular analysis for detection of *EGFR, ALK*, and *ROS1* anomalies (367.66€) to targeted NGS analysis (421.23€) and conclude that NGS could be implemented to routine diagnosis at reasonable costs.

When tumor tissue specimens are insufficient, not contributive, or not obtainable, molecular analyses are conducted on liquid biopsies. Usually, a liquid biopsy corresponds to circulating tumor DNA (ctDNA), which represents only a small proportion (<1%) of circulating free DNA (cfDNA). Discriminating ctDNA from normal cfDNA is aided by the fact that tumor DNA is defined by the presence of somatic mutations. Recently, the development of sensitive and accurate assays has facilitated the detection and quantification of rare variants among a large excess of normal sequences. At this time, the commercially available Cobas *EGFR* mutation test is the only technology approved by the United-Stated (US) Food and Drug Administration (FDA) for the molecular analysis of liquid biopsy specimens in NSCLC. Meanwhile, several digital genomic technologies have been validated for clinical use (38, 39). While these methods exhibit very high sensitivity (around 0.01%), they remain single-gene approaches. New NGS developments are particularly focused on molecular barcodes called unique molecular identifiers (UMIs) and improved bioinformatics pipelines, able to discriminate mutations with a very low allele frequency from sequencing background (40). The MOSCATO trial has demonstrated a good concordance between tumor biopsies and cfDNA NGS analyses (41). This study also demonstrate that molecular diagnosis (comprising tumor biopsy, dispatch of biological samples, histological control, CGH array, targeted NGS, bioinformatics analysis and multidisciplinary molecular tumor board) represent a modest part (6%) of the overall cost of a molecular-guided therapy (42).

### Tools for Detecting Oncogenic Fusions

**Figure 1:**
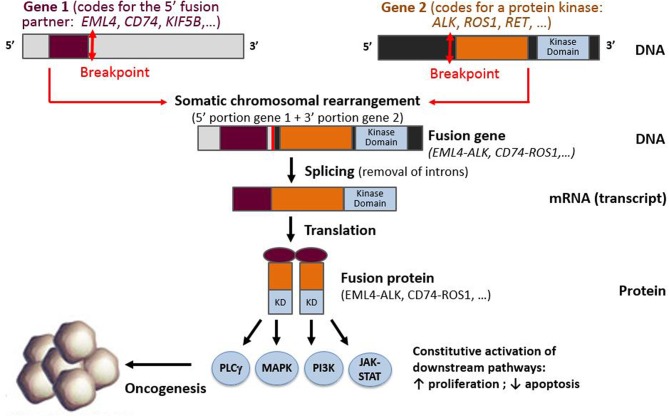
Schematic representation of a fusion between the 3′ portion of a protein kinase gene (containing the kinase domain) and 5′ part of a fusion partner gene, resulting in the production of a fusion gene, fusion transcript, and fusion protein.

In NSCLC, kinase genes, such as *ALK, ROS1, RET, NTRK1*, and *NTRK3* [but also *BRAF, MET, EGFR*, and *fibroblast growth factor receptor* (*FGFR*)] or other proto-oncogenes such as *neuregulin 1* (*NRG1)*, may all be subject to gene rearrangements that lead to constitutive downstream pathway activation (3, 6, 11, 43–45). Since 2007, a wealth of literature exploring *ALK* rearrangements has shown that the detection of gene fusions can prove challenging, because the genomic rearrangements leading to these fusions can be either intra- or interchromosomal (3). In addition, these *ALK* rearrangements have been shown to have multiple partner genes (46–49). In these somatic rearrangements, the 5′ portion of a gene that is expressed by the tumor cell progenitor is fused to the 3′ portion of the proto-oncogene. If an in-frame fusion gene is formed, it is then transcribed into an mRNA fusion transcript that encodes a fusion protein. In the case of kinase gene rearrangements, the fusion protein consists of the N-terminus of the fusion partner, generally containing a dimerization domain, fused to the C-terminus portion of the target gene, containing the kinase domain (Figure 1).

Several molecular tools are currently available for identifying oncogenic fusions in tumor specimens, enabling the detection of these fusions at the DNA, RNA, or protein levels:

- FISH allows the detection of gene rearrangements at the DNA level. The most common assays are based on the use of specific break-apart probes, which are located on either side of the breakpoint in the target gene. This technique was initially considered the gold standard for detecting gene rearrangements, and *ALK* FISH was FDA-approved as a companion diagnostic to help identify patients eligible for crizotinib treatment. However, FISH can lead to false-positive results, because some rearrangements detected on the DNA may not produce a fusion transcript. This approach can additionally miss some small intrachromosomal rearrangements, leading to false-negative results (50–55). Moreover, this technique is not able to provide any indication regarding the nature of the fusion variant (i.e., the fusion partner and introns/exons involved in the fusion).
- Reverse-transcription PCR (RT-PCR) allows the detection of fusion transcripts, although a high number of primer pairs is required for it to be able to detect all known fusions. This is associated with a high risk of false-negatives, considering the diversity of fusions possible, and this approach misses the detection of unknown fusions (56, 57).
- Immunohistochemistry (IHC) can be used to detect fusion proteins, provided a sensitive, specific, and clinically validated antibody is available. This approach is based on the assumption that these antibodies are not specifically directed against fusion proteins, but rather toward their active domain. ALK immunohistochemistry (clone D5F3) has received FDA approval as a companion diagnostic tool, and comparable results have been obtained with the 5A4 clone, provided validated methods are employed (56–63). There are currently no approved IHC-based tests for detecting other fusion proteins, though commercial antibodies are available for identifying ROS1 and TRK proteins.
- NGS, or massive parallel sequencing, assays represent a multiplexed approach to tumor samples' molecular characterization. Considering the high number of molecular alterations described so far in NSCLCs, these assays are likely to experience a bright future in the clinical setting. NGS assays performed on either tissue DNA or RNA enable full characterization of the genes involved in the fusion (rearranged gene and partner gene). However, some of the DNA panels currently available are not designed to detect all possible fusions. Indeed, some panels display only partial intron coverage, especially for genes containing large introns, such as the NTRK genes, or introns with repetitive regions. This can lead to false-negative results, because most breakpoints occur within introns. As with FISH, DNA-based NGS can generate false-positive results, given that some gene rearrangements detected on DNA are unlikely to produce a fusion transcript (50, 64). Conversely, Davies and colleagues recently compared DNA-based and RNA-based NGS assays on 14 ROS1-positive samples. In their study, these authors pointed out that the genomic breakpoint was an unreliable predictor of the transcript-level breakpoint. Moreover, some DNA breakpoints predicted to yield an out-of-frame transcript by DNA-sequencing actually produced an in-frame transcript that was detected by RNA-sequencing (47). Using RNA-based NGS assays for detecting gene rearrangements thus appears to be preferable, because these techniques allow for detecting fusions within the coding regions, in addition to fusions that are likely to result in an expressed fusion protein. This is, however, only applicable if the extracted RNA is of sufficient quality and quantity, which is, undoubtedly, the most critical limitation of these assays (56, 65, 66). Last but not least, NGS approaches permit the full characterization of the introns/exons involved in the fusions, which could provide essential information for the clinicians, if specific fusion variants are found to display different biologic and clinical features, as previously proposed in ALK- or RET-rearranged lung cancers (67–69).

Gene rearrangements are rare events, and no single detection technique has been shown to be 100% sensitive and specific. However, NGS platforms present several key advantages over FISH, IHC, or RT-PCR, given that these platforms not only allow for multiplex testing of both point mutations and rearrangements, but additionally aid molecular characterization of the fusion variant produced. This kind of information could later gain clinical relevance, if some variants prove to be superior predictors of response than others (56, 65, 70–74).

## Therapeutics and Resistance

### EGFR

Since 2009, several first-line Phase III trials have revealed the impressive clinical activity of first- (gefitinib and erlotinib) and second- (afatinib) generation EGFR-TKIs over platinum-doublet chemotherapy, for advanced NSCLC *EGFR*-mutated patients (75–82). Despite the lack of proven evidence regarding OS benefits, probably due to the high percentage of crossed-over patients, these first- and second-generation EGFR-TKIs are clearly associated with a significant benefit in terms of PFS and ORR, namely 9–14 months and 60–70%, respectively. Dacomitinib, a second-generation EGFR-TKI, was investigated in the Phase III ARCHER 1050 trial. This agent was shown to significantly improve both PFS (9.2 vs. 14.7 months, HR 0.59; *p* < 0.0001) and OS (34.1 vs. 26.8 months, HR 0.76; *p* = 0.0438) compared to gefitinib (83, 84). Nevertheless, all four drugs received FDA approbation in the first-line setting, without any distinction made in terms of patient selection (Table 3).

**Table 3:** *ALK* inhibitors used in the first-line setting.

**TKI**	**Brigatinib**	**Crizotinib**	**Alectinib**	**Ceritinib**
Trial	ALTA-1L (85)	ALEX (86, 87)	ALEX (86, 87)	ASCEND-4 (88)
Comparators	Crizotinib	Alectinib	Crizotinib	Platinum-based doublet
N	137	151	152	189
Median PFS (months)	NR	10.4	34.8	16.6
PFS HR (95% CI)	–	–	0.5 (0.36–0.7)^**^	0.55 (0.42–0.73)^*^
ORR (%)	71	75.5	82.9	72.5
Median PFS MC+ (months)	NR	7.4	NR > 27	10.7
Intra cranial ORR (%)	78	50	81	72.7

*ALK, anaplastic lymphoma kinase; TKI, tyrosine kinase inhibitor; N, number of patients; PFS, progression-free survival; HR, hazard ratio; CI, confidence interval; ORR, objective response rate; MC+, patients with central nervous system metastases; NR, not reached*.

* *vs. chemotherapy*.

** *vs. crizotinib*.

Despite an initial response to EGFR inhibitors, patients are most likely to develop acquired resistance and relapse after 8–13 months of treatment (89). Several mechanisms have been proposed to account for this. The most common (50–60% of cases) is the T790M mutation in exon 20, which changes the conformation of the protein and prevents TKIs' binding to the ATP pocket (90, 91). Another resistance mechanism is the activation of bypass signaling pathways, such as HER2 or MET (92, 93), as well as EGFR downstream signaling pathways (94). Additionally, phenotypical transformations, such as SCLC transformation and epithelial mesenchymal transition (EMT), have been described as resistance mechanisms to EGFR-TKIs (95, 96).

**Figure 2:**
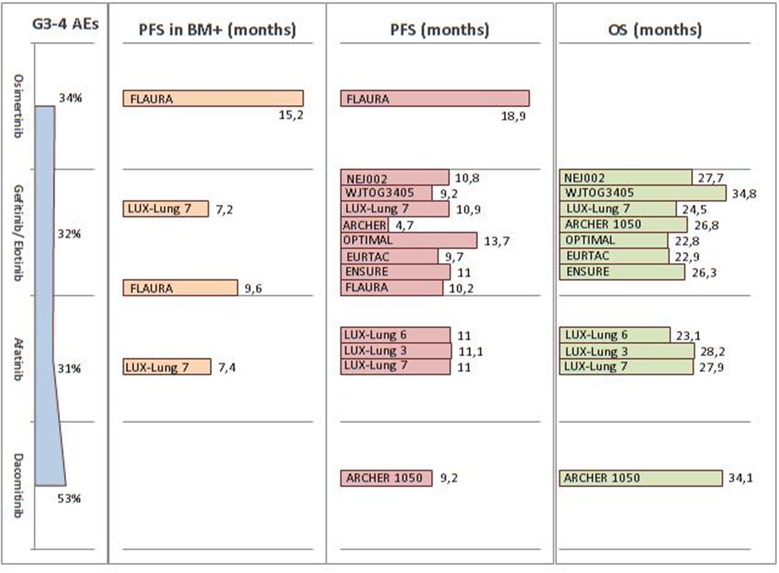
Summary of FDA and EMA approved EGFR-TKI efficacy and tolerability across Phase III trials in the first-line setting. FDA, Food and Drug Administration; EMA, European Medicines Agency; EGFR-TKI, epidermal growth factor receptor tyrosine kinase inhibitors; AE, adverse event; PFS, progression-free survival; OS, overall survival; BM, brain metastasis.

Osimertinib, a third-generation EGFR-TKI, was recently approved as either first-line agent for metastatic and locally advanced EGFR mutant patients or second-line drug for those presenting the T790M resistance mechanism (97). In the Phase III FLAURA trial, osimertinib nearly doubled the median PFS, compared to erlotinib or gefitinib (18.9 vs. 10.2 months, HR 0.46; *p* < 0.001). It should, however, be noted that the OS data were not yet mature at analysis (98). Considering these crowded scenarios, the first-line choice should primarily be driven by the patient's characteristics and tolerability profile. In the FLAURA trial, osimertinib had a lower rate of Grade 3–4 undesirable events compared to first-generation EGFR-TKIs (34 vs. 45%). Furthermore, the hazard ratio for PFS was similar in patients either with or without known brain metastasis (0.47 and 0.46, respectively). These data have been summarized in Figure 2.

Despite the impressive efficacy of third-generation EGFR TKIs in T790M positive NSCLC, acquired resistance invariability is likely to occur via several, though only partially-known, mechanisms. These resistance mechanisms can be divided into three categories: 1) reactivation of the EGFR pathway through *EGFR* novel mutations; 2) activation of bypass signaling pathways; 3) histological shift able to bypass the EGFR-dependence in form of or small-cell lung cancer (SCLC) transformation (99–101). At resistance, around 50% of patients were demonstrated, across different series, to maintain the T790M mutation. Novel *EGFR* mutations represent the most commonly acquired resistance mechanism in this group, while the C797S mutation accounts for 22–40% of all progressions under third-generation EGFR-TKIs (100, 102). Interestingly, cell lines harboring dual C797S and T790M alteration in *trans* were sensitive to a combination of first- and third-generation EGFR-TKIs, as opposed to a mutation in *cis* (103). Conversely, patients with a loss of T790M alteration at resistance more frequently presented an *EGFR* bypass alteration, either by downstream EGFR (RAS-MAPK pathway) signaling or activating parallel signaling pathways, such as MET or ERBB2 amplifications, PIK3CA mutations, and PTEN loss (99–101). These data highlight the urgent need for investigative or combination therapies to effectively prevent or treat the drug resistance complexity in EGFR-mutant lung cancer.

Other uncommon alterations (exon 18 G719, exon 21 L861Q, and exon 20 S768I) are recognized as sensitizing EGFR-TKIs, with different magnitudes of response. In a large cohort of *EGFR*-mutated patients treated with first-generation EGFR-TKIs, ORR was significantly lower in patients with uncommon alterations, compared to classical *EGFR* mutations (47.5 vs. 74.1%; *p* < 0.001) (104). Other studies have similarly reported data concerning the effectiveness of EGFR-TKIs on these rare mutations. These include a combined *post-hoc* analysis of LUX-Lung 2, LUX-Lung 3, and LUX-Lung 6 (105), in addition to the Phase II trial KCSG-LU15-09 (106). Among the upcoming target agents, poziotinib demonstrated promising activity in a Phase II trial that enrolled pretreated NSCLC patients with *EGFR* exon 20 mutation (excluding acquired T790M). This resulted in 55% ORR and 5.5 months mPFS, along with a high rate of Grade 3–4 undesirable events (56%) (107).

### ALK

Five ALK inhibitors are currently available: first generation (crizotinib), second generation (alectinib, ceritinib, and brigatinib), and third generation (lorlatinib). These therapies have dramatically changed patients' prognosis, as they have improved the median OS from diagnosis made in the metastatic stage. Indeed, the strategy using sequencing ALK inhibitors has resulted in a median 81 months OS in two recent studies (108, 109).

Crizotinib, a molecule-targeting MET, ROS1, and ALK, was the first TKI to generate promising results in *ALK*-positive patients (30). Compared to standard chemotherapy in ALK-positive advanced NSCLC, crizotinib demonstrated a longer median PFS (10.9 vs. 7 months; HR 0.45), along with a higher ORR (74 vs. 45%) (110). After crossover adjustments, OS was increased in the crizotinib arm (HR 0.346) (111). Compared to chemotherapy, crizotinib was found to be well-tolerated, primarily causing color vision disorders, digestive disorders, or edema. Ceritinib was shown associated with a doubled median PFS (16.6 months), in comparison to chemotherapy (8.1 months) (HR 0.55) (88). In this Phase 3 study, among 22 patients with brain metastases, the intracranial ORR was 72.7% in the ceritinib group and 27.3% in the chemotherapy group. The most common undesirable events of ceritinib included digestive disorders and increased alanine aminotransferase levels. The ALEX trial, which compared alectinib to crizotinib in the first-line setting, has proven an higher alectinib efficiency, with a comparable safety profile (86, 87). Indeed, the median PFS in the alectinib arm was 34.8 months vs. 10.9 in the crizotinib arm (HR 0.43). Furthermore, alectinib exhibited a better cerebral activity, with a median PFS of 27.7 months in patients with central nervous system (CNS) metastases at baseline, compared to 7.4 months for the crizotinib group (HR 0.35; 95% CI: 0.22–0.56). Alectinib was shown to be associated with a lower incidence of CNS progression (four times less) in patients without CNS metastasis at diagnosis, compared to crizotinib. Considering its safety profile, alectinib primarily induced myalgia, anemia, and liver disorders. Finally, in the first-line setting, brigatinib has been proven superior over crizotinib, in terms of efficacy (85). Indeed, the 12-months PFS in the brigatinib group was higher (67%) than in the crizotinib arm (43%), with statistically significant results (HR 0.49; 95%CI: 0.33–0.74; *p* < 0.001). Likewise, brigatinib was associated with an intracranial ORR of 78%, compared to 29% in the crizotinib group. Furthermore, their safety profiles were comparable. The most common undesirable events of brigatinib conprised digestive disorders, hypertension, and increased alanine aminotransferase ALAT and lipase levels. Lastly, lorlatinib has been tested in a Phase II study that enrolled 228 *ALK*-positive patients, with or without previous ALK-TKI or chemotherapy exposure (112). Lorlatinib showed an ORR of 90% in the treatment-naïve group (30 patients) and of 47% beyond the first-line setting (198 patients). Likewise, an intracranial response was observed in 66.7% of the treatment-naïve patients (*n* = 3) and in 63% of previously-treated patients (*n* = 81). Lorlatinib was found to induce dyslipidemia, peripheral neuropathy, and mood or cognitive effects.

However, ALK inhibitors have been shown to consistently cause the development of resistance mechanisms. After a first ALK-inhibitor, treatment choice can be guided by a new sample with the identification of an *ALK*-resistance mechanism (113). These mechanisms can be either *ALK*-dependent or ALK-independent. ALK-dependent mechanisms correspond to secondary mutations in the ALK kinase domain. These mutations induce kinase and signaling reactivation by preventing the binding of TKIs to their targets. These mutations occur in 20–30% of patients treated with crizotinib, and in 50–70% of patients after second-generation ALK inhibitor exposure (113). With second-generation inhibitors, the highly resistant G1202R mutation was shown to be more common, which then requires initiating the third-generation TKI lorlatinib (114, 115). Indeed, an *in vitro* study has shown different sensitivity profiles of ALK inhibitors, with respect to various resistance mutations (113). *ALK* amplifications were revealed to be less frequent than *ALK* mutations, most commonly occurring following crizotinib therapy. ALK-independent mechanisms consist of the activation of bypass signaling pathways, such as EGFR activation, *MET* amplification, MEK reactivation, or phenotypic changes like EMT or SCLC transformation (25, 116–119). An overexpression of P-glycoprotein (PGP), an efflux pump, was additionally identified as a potential resistance mechanism (114). PGP was shown to be responsible for decreased ALK inhibitor (crizotinib or ceritinib) concentrations in brain tissues (120, 121).

### MET

The *MET* gene encodes a transmembrane receptor that is normally activated by the binding of its ligand, the hepatocyte growth factor (HGF). MET pathway activation is thought to occur through diverse mechanisms that influence properties affecting cancer cell survival, growth, and invasiveness (122). Somatic dysregulation at MET occurs through several different mechanisms that are non-exclusive in NSCLC, including protein overexpression, gene amplification, mutation, and rearrangement. *MET* amplification was revealed to be uncommon in previously-untreated NSCLC patients and was found in about 2–4% of cases. *MET* amplification appears to play a role in acquired resistance to EGFR inhibitors and is observed in about 5–20% of patients in this setting (12, 123, 124). *MET* alterations that result in exon 14 skipping were observed in about 3–4% of NSCLCs (12, 125). *METex14* mutations were more frequently identified in adenocarcinoma and sarcomatoid carcinoma patients. These mutations were found more frequently in older rather than younger patients. Of note is that these mutations were revealed to be mutually exclusive to other driven mutations (126).

Several clinical trials have been carried out so far, but most of them did not produce positive results (127–129). Preliminary data on capmatinib revealed encouraging results in patients with *METex14* mutations in either previously-treated or treatment-naïve NSCLC patients. The GEOMETRY trial showed a clinically meaningful ORR of 39.1% and 71.4% in previously-treated and treatment-naïve patients, respectively, with a manageable toxicity profile (130). Tepotinib, a MET-selective TKI, generated promising activity in *METex14* patients, with an ORR of 59%. In terms of safety, more than 50% of patients experienced tepotinib-related treatment-emergent adverse events (TRTEAEs), including serious TRTEAEs in three cases (8.8%) (131). Crizotinib obtained FDA breakthrough designation in *METex14* NSCLC, based on the results of an expansion cohort from the Phase I PROFILE 1001 study, which included 69 *METex14* patients. Among 65 evaluable patients, there were three complete responses (4.6%), 18 (27.7%) partial responses, and 29 stable disease cases. Median time to response was 7.6 weeks, with a median duration of response (DOR) of 9.1 months and PFS of 7.3 months (132). The AcSé program enrolled 25 patients with *MET* amplification and 29 with *MET* mutation (25 *METex14*). Crizotinib showed activity in both *MET*-amplified and *MET*-mutated NSCLC (ORR: 32 and 40%, respectively) patients, whereas response correlated with the number of *MET* copies in the amplified group. Median PFS was around 3.5 months in both groups, while OS was longer in the METex14 population (9.5 vs. 7.7 months) (133).

### BRAF

*BRAF* (B-Raf proto-oncogene, serine/threonine kinase) base substitutions are present in approximately 2–5% of NSCLCs; about half of these mutations result in V600E amino acid substitution (13, 134, 135). The *BRAF* mutation induces the activation of the MAPK pathway, promoting cell growth, proliferation, and survival. Gene fusions that are biologically distinct from V600E mutations have been identified more rarely, in 0.2% NSCLC cases (136). Most patients with *BRAF* mutations were revealed to be former smokers, whereas non-V600E mutations were more commonly found in heavy smokers (44).

Preliminary data, pertaining to a single-agent BRAF inhibitor like dabrafenib or vemurafenib, consisted of a 30–40% ORR and 5–7 months median PFS (137, 138). The most interesting results were, nevertheless, obtained when combining dabrafenib with the MEK inhibitor trametinib. A Phase 2 trial in chemotherapy-pretreated NSCLC patients generated an OS of 18 months, compared to 12.7 months observed under dabrafenib monotherapy (139). Another Phase 2 trial in BRAF(V600E)-positive, chemo-naïve patients confirmed this combination's efficacy, reflected by a 64% ORR, along with a PFS and OS of 11 and 25 months, respectively (140). Based on these results, both the FDA and European Medicines Agency (EMA) approved in 2017 the combination of dabrafenib and trametinib for patients with advanced NSCLC harboring a V600 mutation, regardless of the therapy line.

### ROS1

*ROS1* rearrangements are detected in approximately 1–2% of lung adenocarcinomas (43). In Europe, *ROS1*-rearrangement testing is recommended in never smokers with advanced *EGFR/KRAS/ALK*-negative NSCLC. In current and former smokers, ROS1 testing is indicated only in non-squamous histology (141). The high amino-acid sequence homology between ROS1 and ALK kinase domains explains the significant clinical activity of crizotinib in both ROS1 and ALK-driven tumors. Indeed, in the expansion cohort of the Phase I trial PROFILE 1001, 50 ROS1-positive NSCLCs received crizotinib, reaching an ORR of 72% and a median PFS of 19.2 month (142). Median OS was 51.4 months and no correlations were observed between overall survival and specific *ROS1* fusion partners (143). Several Phase II trials conducted on European and Asian cohorts showed similar results, with reported ORR of about 70% and PFS ranging from 10 to 13 months (144–146). As with other oncogene-addictions, mechanisms of acquired resistance also occur in ROS1-positive NSCLC cancers, mainly through mutations in the target itself or via activation of alternative pathways. Several molecules have shown promising results in overcoming resistance to crizotinib in ROS1+ NSCLCs. For example, ceritinib showed an ORR of 62% and a median PFS of 9.3 months in a phase II trial conducted on 28 pretreated or untreated patients with advanced *ROS1*-rearranged NSCLC. The median PFS was even longer (19.3 months) for crizotinib-naïve patients and the median overall survival (OS) was 24 months (147). Alectinib and brigatinib, have little to no established ROS1 inhibitory activity. By contrast, lorlatinib also appears to be able to overcome acquired resistance to crizotinib in ROS1-positive NSCLC, including disease in the CNS (112). Newer ROS1 inhibitors are in development, and investigational agents such as cabozantinib, entrectinib, repotrectinib, and DS-6051b have emerging results in early-phase clinical trials in *ROS1*-rearranged NSCLC (148–150).

### NTRK

The *NTRK* genes, consisting of *NTRK1, NTRK2*, and *NTRK3*, encode the tropomyosin receptor tyrosine kinases TRKA, TRKB, and TRKC, respectively, which function during normal neuronal development and maintenance (151–153). While the frequency of *NTRK* fusions proves to be low in common cancer types, including NSCLC, *NTRK3* fusions were revealed to be almost ubiquitous among rare cancer types, such as mammary analog secretory carcinoma and infantile fibrosarcoma (44). In NSCLC, *NTRK* fusions are estimated to occur at a frequency of approximately 0.1 to 1% (7, 44), whereas the clinical and pathologic features of the patients harboring such fusions have not yet been well-characterized.

Considering the rarity of these fusions, drug development has been primarily derived from basket trials. Among them, larotrectinib (LOXO-101) and entrectinib (RXDX-101) produced promising results, in fusion-positive NSCLC patients (132, 154). A Phase 1 study involving adults only, Phase 1–2 study involving children only, and Phase 2 study involving both adolescents (*n* = 55) and adults treated with larotrectinb showed an ORR of 75%, with the median DOR and PFS not yet reached at data cut-off. Larotrectinib tolerability was acceptable, with predominantly Grade 1 undesirable events reported (132). Entrectinib, an oral TKI of TRKA/B/C, ROS1, and ALK, was evaluated in two Phase 1 studies. These trials revealed a good toxicity profile and rapid and durable responses in patients with brain metastases (154). A Phase 2 trial with entrectinib in solid tumor patients harboring *NTRK1/2/3, ROS1*, or *ALK* gene rearrangements is still ongoing (NCT02568267), as are several other trials involving TKIs against TRKA/B/C, including TSR-011 (NCT02048488), DS-6051b (NCT02279433), and PLX7486 (NCT01804530).

### RET

The *RET* (rearranged during transfection proto-oncogene) fusion oncogene and fusions proteins resulting from chromosomal rearrangement were initially described in thyroid carcinoma patients (155). *RET* fusions were subsequently identified in approximately 1–2% of NSCLC patients; it was found to be mutually exclusive with other oncogenic drivers (5, 6, 11, 156, 157). At least 12 different *RET* gene partners have been described, the most common being *KIF5B* (70% of cases), followed by *CCDC6* (20% of cases) (48). Data from the Global Multicenter RET registry did not reveal any significant difference in incidences between men and women, whereas the majority of affected patients had never been smokers (63%) and suffered from a metastatic adenocarcinoma (48).

In NSCLC, many multi-targeted kinase inhibitors have been tested to date. These include cabozantinib, vandetanib, sunitinib, sorafenib, alectinib, lenvatinib, nintedanib, ponatinib, and regorafenib. Yet, their activity was shown to be clearly inferior to the responses and survival outcomes seen with selective TKIs in other oncogene-addicted NSCLC models (48, 158, 159). To date, no drugs have been approved for RET-rearranged NSCLCs (126). New RET selective inhibitors are, however, in development (e.g., LOXO-292 and BLU-667). LOXO-292 was found to generate encouraging clinical activity in RET-altered solid tumors in a Phase 1 trial, with a 77% ORR for NSCLCs patients, including those with brain metastases. At data cut-off, the median DOR was not yet reached, while the longest response already exceeded 10 months (160, 161). Based on these data, further studies are needed to further confirm this drug's potential benefit.

## Conclusion

In advanced NSCLC, identifying molecular alterations proves to be a daily challenge. Tumor samples can be either tissues with low tumor cellularity or liquid biopsies. Biologists increasingly use efficient techniques to identify these oncogenic alterations. NGS platforms, therefore, occupy a central place. Other promising techniques are also emerging, such as radiogenomics, for example, which combine non-invasive imaging and molecular analysis, study the link between genomic and phenotypic information. These techniques could help for the continuous monitoring of patients with advanced NSCLC and to follow their treatment responses (162).

For patients with *EGFR* mutations or *ALK/ROS1* rearrangements, several TKIs are currently available, designed either for front-line therapy or patients at progression. These TKIs are more effective than chemotherapy, well-tolerated and remain the reference treatment. For the other oncogenic alterations (*MET, BRAF, NTRK*, and *RET*), while several new molecules appear promising, further studies are still required. Concerning immune checkpoint inhibitors, according to a recent study, these molecules have not demonstrated a better efficacy and should be used after TKIs and chemotherapy failures, especially in *ALK*-positive NSCLCs (163).

As a wealth of diagnostic tools and personalized treatments are already available or still under development. A close relationship between molecular biologists, pathologists, and oncologists is crucial.

## Author Contributions

JP, AM-F, MG, FF, CE, EG, and A-CT wrote/edited the manuscript and approved it for submission.

### Conflict of Interest

JP reports personal fees or grants from MSD, Pierre Fabre, Roche, Pfizer, and Takeda. AM-F reports personal fees from Pfizer, Takeda, AstraZeneca, and Boehringer, research grants from Pfizer, Novartis, and Takeda, and non-financial support from Roche, Clinisciences, AstraZeneca, Takeda. MG reports personal fees from BMS, Roche, Novartis, MSD, and Astra Zeneca, as well as grants from BMS. FF reports personal fees from Roche diagnostic and AstraZeneca. EG reports personal fees from Astrazeneca, grants and personal fees from Bristol-Myers Squibb, personal fees from Roche, and personal fees from Merck Sharpe and Dohme, outside the submitted work. A-CT reports grants, personal fees, and non-financial support from Roche; grants, personal fees and non-financial support from AbbVie; personal fees and non-financial support from MSD; personal fees and non-financial support from BMS; personal fees and non-financial support from Boehringer Ingelheim; personal fees and non-financial support from Astra Zeneca; personal fees from Vifor Pharma; personal fees from Novartis. The remaining author declares that the research was conducted in the absence of any commercial or financial relationships that could be construed as a potential conflict of interest.
